# Research on the influencing factors of users’ continuance intentions of wearable medical devices—a perspective integrating the UTAUT model and gamification elements

**DOI:** 10.3389/fpubh.2025.1602230

**Published:** 2025-07-18

**Authors:** Yulin Tian, Zoumin Li, Dong Liu, Sangbum Son

**Affiliations:** ^1^School of Business, Shandong Xiehe University, Jinan, Shandong, China; ^2^School of Business Administration, Shandong Women’s University, Jinan, China; ^3^Department of Global Business, Yeungnam University, Gyeongsan, Republic of Korea

**Keywords:** wearable medical devices, gamification, UTAUT model, users’ continuous usage intention, structural equation modeling

## Abstract

With the global aging population and the increasing prevalence of chronic diseases, the application of wearable medical devices in health management has garnered widespread attention. However, despite the significant advantages of these devices in health monitoring and disease prevention, many users discontinue their use within a short period, posing challenges to their long-term adoption. This study develops a research framework by combining the Unified Theory of Acceptance and Use of Technology (UTAUT) model with gamification components to methodically examine the critical factors influencing users’ sustained intention to use wearable medical devices (WMD). A survey method was employed, collecting 362 valid responses, and applied structural equation modeling (SEM) for empirical analysis. Results demonstrate that performance expectancy, effort expectancy, social influence, and gamification elements all exert a significant positive effect on users’ continued usage intention. The results contribute to the theoretical foundation for improving the design and market promotion of WMD and offer actionable recommendations for developers and policymakers to boost user adoption.

## 1 Introduction

In recent years, with the intensification of population aging and the trend of diseases occurring at younger ages, the prevalence of chronic diseases has significantly increased, drawing widespread social attention to health issues. Numerous studies have shown that unhealthy lifestyle behaviors such as sedentary habits (1), obesity (2), and poor sleep quality (3) are directly related to the onset of various chronic diseases (4). Against this backdrop, the growing demand for health management has become an inevitable trend in social development (5).

However, the uneven distribution of medical resources restricts access to timely and effective healthcare services for certain populations, particularly residents of remote areas or individuals with mobility impairments (6, 7). As a result, how to leverage advanced technologies to enhance personal health monitoring and management capabilities has become an important research topic in the field of health. Wearable medical devices (WMD) have seen widespread adoption in recent years, providing innovative solutions for health monitoring. Research has confirmed that these devices can offer continuous health monitoring, data tracking, and physiological parameter measurement, demonstrating significant value in health management (4, 6, 8). With tech companies like Apple, Samsung, and Xiaomi entering the healthcare sector, smart WMD such as smartwatches, smart bands, and smart rings have quickly become popular (9). These devices not only offer portability and wearability (10) but also integrate the functionality of smartphones, providing users with real-time feedback on health data and personalized health management services (11, 12). This enables users to contact doctors in a timely manner and provides necessary data for medical interventions, thus reducing health risks (5, 6, 8). However, despite the numerous advantages of WMD in health management, studies have found that approximately 30% of users abandon them after short-term use, suggesting that, after the novelty wears off in the initial phase, users may reduce their willingness to use the devices due to a lack of sustained motivation (13, 14). Therefore, increasing users’ willingness to continue using WMD has become a central issue in current research.

In recent years, gamification has emerged as a powerful strategy for improving user experience and fostering positive behavioral intentions (15), finding extensive application in diverse domains, including marketing (16), education (17), online banking (18), and mobile payments (19). However, in the field of WMD, research on how gamification influences users’ continuous usage behavior remains limited, which provides significant theoretical potential for this study to explore.

To address this research gap, this study will combine the Unified Theory of Acceptance and Use of Technology (UTAUT) model to explore the mechanism by which gamification affects users’ intention to continue using WMD. The UTAUT model, due to its integration of factors from multiple classic technology acceptance models, has strong theoretical explanatory power in explaining user behavior intentions (20). In the field of WMD, prior research has utilized the UTAUT model to examine older adult users’ adoption intentions (21). Additionally, Venkatesh et al. (22) argued that the UTAUT model could be further enhanced by introducing new variables or boundary conditions. For example, some scholars have combined the UTAUT model with gamification to explore the adoption of mobile health apps (23). Accordingly, this study incorporates gamification as an extended variable into the UTAUT model, aiming to provide a novel perspective for understanding the mechanisms that influence users’ continued use of WMD.

Based on the context provided, this research aims to explore and answer the following research question.

*RQ1*: How does gamification influence users’ intention to continuously use WMD?

This study will utilize the UTAUT model to examine the relationship between gamification and users’ continuous usage behavior. This study is structured as follows: The first section offers a theoretical review of WMD, the UTAUT model, and gamification theory. Building on the theoretical framework, the second section develops the research model and proposes hypotheses. The third section conducts a survey and data analysis. In the fourth section, the research findings are analyzed, their theoretical and practical implications are explored, and suggestions for future research are proposed.

## 2 Literature review and theoretical background

### 2.1 Wearable medical devices

The WMD are smart electronic devices connected to mobile internet that can monitor users’ health conditions in real time (24). These devices integrate computer technology with portable design and can be worn on the body to support health monitoring and management. WMD offer several notable advantages. First, these devices provide users with critical physiological indicators (such as blood oxygen levels, heart rate, and blood pressure) and environmental information (such as noise levels) anytime and anywhere while also tracking behavioral patterns (such as sleep quality and step count) (13). This capability enables users to stay informed about their health status in real time, facilitating early disease prevention. Second, WMD enhance communication efficiency between doctors and patients. By collecting and transmitting data, these devices allow doctors to gain a more accurate understanding of patients’ health conditions, reducing unnecessary medical consultations and improving the quality of telemedicine services (25, 26). Third, the use of these devices can significantly reduce the frequency of hospital visits, thereby alleviating the burden on public healthcare resources (13). Additionally, they help lower the risk of patients being exposed to infectious diseases in hospital settings, providing more comprehensive protection for public health (27, 28).

### 2.2 UTAUT model

Proposed by Venkatesh et al. (20), the UTAUT model is a unified framework derived from the integration of eight well-established technology acceptance models. The goal is to uncover the critical factors and mechanisms that drive the acceptance and adoption of new technologies. The model is structured around four primary constructs: performance expectancy (PE), effort expectancy (EE), social influence (SI), and facilitating conditions (FC).

Due to its strong theoretical basis and practical significance, in the healthcare sector, the UTAUT model has been extensively utilized. For instance, studies have shown that the UTAUT model effectively explains the adoption of mobile health services (29), the utilization of health information technology (30), and the advancement of electronic health records (31). Additionally, in the field of wearable devices, scholars have also conducted in-depth studies based on the UTAUT model. For example, Chen et al. (6) analyzed the effects of PE, perceived cost, hedonic value, and aesthetic preferences on older adults’ inclination to adopt wearable devices. By incorporating the UTAUT model and task-technology fit theory, Wang et al. (13) examined the behavioral mechanisms driving consumer adoption of healthcare wearable devices.

Leveraging the UTAUT model’s theoretical foundation and wide applicability, the aim of this study is to determine the core factors that affect users’ ongoing intention to use WMD. These factors include PE (users’ perceptions of the effectiveness and practicality of using WMD for health monitoring and management), EE (the time and effort users perceive they need to invest in using WMD), SI (the behavioral expectations users feel from significant others or social groups), and FC (whether users receive adequate technical support, device resources, and environmental guarantees when using WMD). These factors will enhance our understanding of the behavioral mechanisms driving users’ continued use of WMD and offer theoretical guidance for optimizing device design and developing effective marketing strategies. Additionally, by incorporating new variables such as gamification, this study will extend the application of the UTAUT model, offering a fresh perspective for research and practice in the healthcare technology field.

### 2.3 Gamification

There are two main perspectives on the definition of gamification in the academic literature. The first perspective, introduced by Deterding et al. (32), defines gamification as the application of game design elements and principles to non-game settings. The second perspective, proposed by Huotari and Hamari (33), defines gamification, from a service marketing standpoint, as the process of enhancing the service value in a non-game environment by providing users with a gamified experience. In this study, within the context of health management using WMD, gamification is defined as the process of offering users a more engaging and interactive health management service experience through the integration of game elements like points, badges, and leaderboards.

Empirical studies have consistently validated the positive impact of gamification across a wide range of domains. In the field of marketing, gamification is recognized as an important strategy for enhancing brand engagement (16). Within the context of mobile applications, it has been shown to be an effective mechanism for increasing consumer shopping engagement (34). In mobile learning environments, researchers have found that gamification fulfills users’ basic psychological needs, thereby improving learning performance (17). In the fintech sector, studies have revealed that gamification significantly influences users’ adoption of mobile payment applications in a positive manner (19). In recent years, gamification has also been increasingly applied to research on the adoption of health-related technologies, demonstrating positive effects particularly in therapeutic settings (35, 36) and physical exercise contexts (37, 38).

Unified Theory of Acceptance and Use of Technology has been widely employed to understand users’ adoption behavior toward emerging technologies (29–31). While the UTAUT model demonstrates strong predictive validity, it remains limited in its ability to account for intrinsic motivational mechanisms—particularly when explaining users’ intentions for continued use. To address this theoretical gap, researchers have begun incorporating gamification into the UTAUT model as an external motivator and contextual trigger, thereby enhancing the model’s explanatory power regarding users’ psychological processes. For example, Katheeri (23) integrated gamification with the UTAUT model to examine user adoption of mobile healthcare applications. Other studies have embedded gamification mechanisms into the UTAUT framework to analyze user adoption in internet banking, confirming the positive moderating effect of gamification on continued usage intention (18). Therefore, in the context of this study, integrating gamification with the UTAUT model offers a more comprehensive understanding of users’ continued intention to use medical wearable devices.

## 3 Research model development and hypothesis formulation

### 3.1 Research model

**Figure 1:**
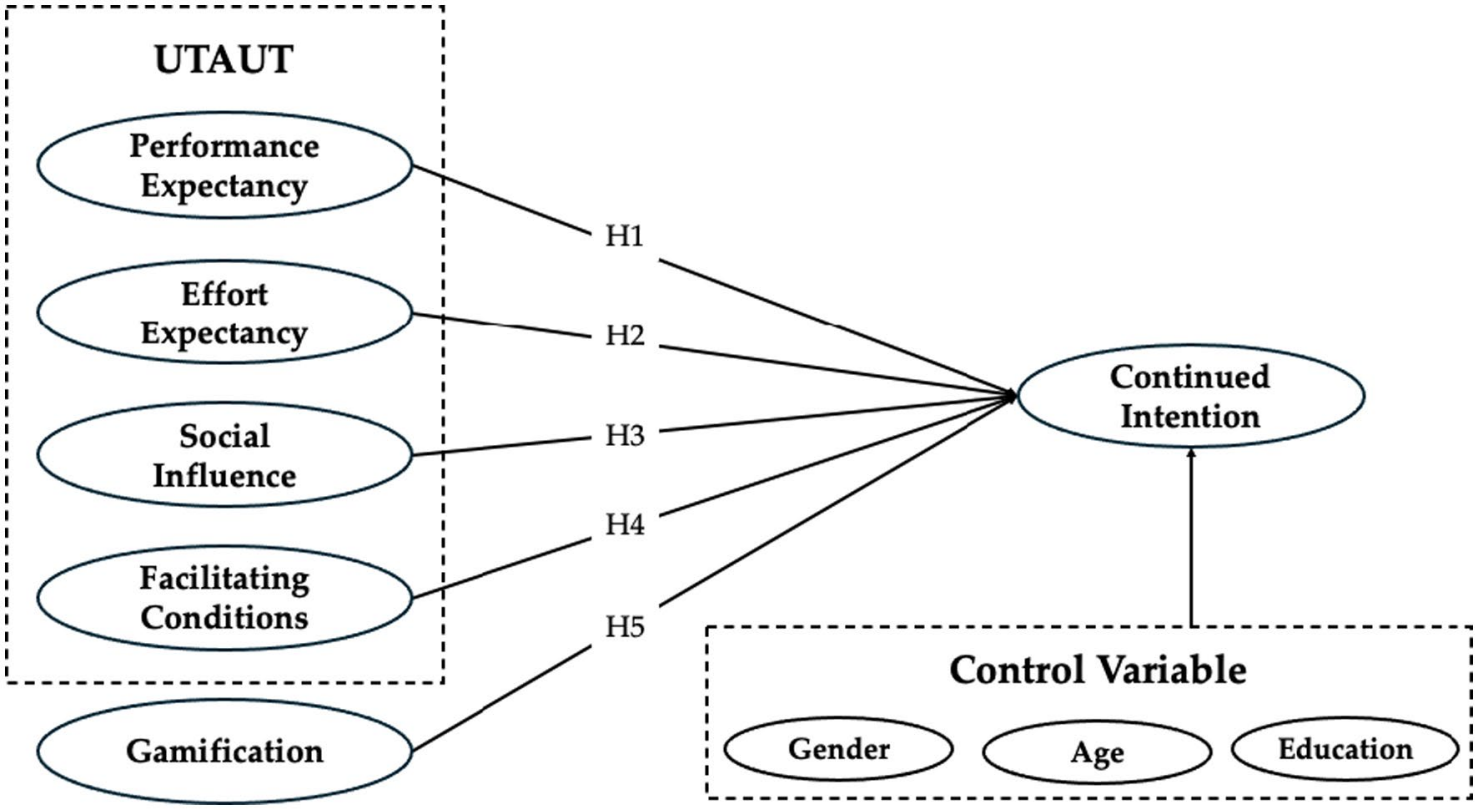
Research model.

To gain deeper insights into the key factors affecting users’ continued willingness to engage with WMD, by integrating gamification as an additional variable, this study extends the UTAUT model, resulting in the development of the following research framework (refer to Figure 1).

### 3.2 Hypothesis development

PE refers to the extent to which users believe that adopting a specific technology will improve their performance in work or daily life (20). A study on food delivery apps found that PE positively influenced users’ continued intention to use the service (39). Within the healthcare sector, PE has also been widely confirmed to significantly influence users’ continued usage intentions. For example, research by Tian and Wu (40) showed that PE positively affected older adult users’ willingness to continue using mobile health services. Furthermore, research examining the continued usage intentions of middle-aged and older adult users toward health maintenance-oriented WeChat official accounts (HM-WOAs) revealed that users were more inclined to sustain their use of these services when they perceived greater health benefits from the accounts (41).

As an emerging medical technology, WMD offer significant advantages. The design of their WMD eliminates limitations of time and space, enabling users to monitor and track real-time daily health metrics such as heart rate, blood pressure, blood oxygen levels, and physical activity data. This real-time monitoring and convenience provide efficient support for users’ health management. Therefore, when users perceive WMD as more efficient and practical for health management, their continued willingness to use the WMD may experience a notable increase. Based on the points outlined above, this study proposes the following hypothesis:

*H1*: Performance expectancy positively influence the intention to continue using WMD.

Effort expectancy reflects the extent to which users perceive a particular technology as user-friendly or requiring little effort to operate effectively (20). In the realm of mhealth services, the need for users to operate medical technologies and monitor data can significantly impact the accuracy and reliability of mobile health services. Previous studies have indicated that EE positively affects users’ behavioral intentions in the mobile healthcare sector (29). Moreover, research has shown that middle-aged and older adult users are more likely to sustain their use of mhealth services when they perceive the healthcare system as easy to use and navigate (40).

In the context of WMD, when users perceive the devices as easy to operate, with an intuitive interface and seamless functionality, the cognitive and operational burden required during use is lower. This significantly enhances users’ intention to continue using WMD. Based on the points outlined above, this study proposes the following hypothesis:

*H2*: EE positively influences the intention to continue using WMD.

The SI refers to the degree to which individuals believe that significant others or social groups expect them to use a particular technology (20). Theoretical research suggests that, in order to gain social approval and enhance self-worth, individuals often engage in behaviors that align with others’ expectations (42). Within the healthcare field, SI significantly impacts nurses’ willingness to adopt electronic health records (31). Additionally, Ahmad et al. (43) highlighted that the perspectives of key individuals in the lives of older adult diabetic patients positively influenced their continued use of WMD. Building on the research background mentioned above, this study suggests that in the context of WMD usage, users’ behavioral intentions will be significantly influenced when they perceive positive recommendations from important others or social groups. Based on the points outlined above, this study proposes the following hypothesis:

*H3*: SI positively influences the intention to continue using WMD.

Facilitating condition refer to the degree to which users believe that the necessary organizational and technological resources are in place to support the implementation and use of a specific technology (20). When users perceive that service providers can offer reliable, continuous monitoring and feedback services, they have a higher likelihood of adopting the technology (44). A study on green financial technologies found that FC positively impacted users’ willingness to continue using the technology (45). Within the realm of mobile healthcare services, FC has been identified as a key determinant of users’ acceptance of mobile health technologies. When users perceive that mobile healthcare services are supported by adequate infrastructure, necessary tools, and technological guarantees, they are more willing to adopt these services (40, 46). Further research in healthcare services indicates that older adult diabetic patients are more willing to use wearable devices long-term if they can be connected to their existing devices, such as smartphones (43).

In the context of wearable medical device usage, users’ perceptions of external resources, tools, and environmental conditions that support the use of the device are particularly critical. If users believe that FC are sufficient—such as convenient technical support, effective after-sales service, or reliable device functionality—they are more likely to continue using WMD for health management. Based on the points outlined above, this study proposes the following hypothesis:

*H4*: FC positively influences the intention to continue using WMD.

Gamification plays a key role in boosting user motivation and increasing the likelihood of continued technology use. In research within the field of human-computer interaction, Wang et al. (47) found that, compared to non-gamified systems, users exhibited higher behavioral intentions toward gamified systems, and they were more likely to recommend gamified systems to others. In studies on fitness applications, Koivisto and Hamari (48) highlighted that the introduction of gamification elements such as point rewards, goal setting, and achievement unlocking significantly increased user engagement and the willingness to continue using the application, while also creating a more enjoyable user experience.

In the context of WMD, gamification elements such as goal tracking, reward systems, achievement unlocking, and leaderboards can make the health management process more engaging and interactive, thereby enhancing users’ sense of experience and participation. By combining health management with entertaining elements, gamified designs may effectively motivate users to continue using WMD to reach their health objectives. Therefore, the following hypothesis is put forward in this study:

*H5*: Gamification positively influences the intention to continue using WMD.

## 4 Empirical research

### 4.1 Survey design and data collection methods

This study, drawing on existing literature and the research background, developed a structured questionnaire to guarantee the measurement tool’s scientific validity and practical relevance. A multi-item approach is adopted in the questionnaire, with each indicator evaluated using a 5-point Likert scale from 1 (“Strongly Disagree”) to 5 (“Strongly Agree”). The specific measurement items are outlined in Appendix Table A1. Building on previous research, these items were adjusted and refined to align with the research context, allowing for a thorough assessment of the factors driving users’ sustained usage of WMD. All survey questions are mandatory to ensure data completeness and validity, meaning respondents cannot submit the questionnaire until all items are completed.

To maintain the questionnaire’s validity, this study first invited scholars and professionals in information management systems to review the questionnaire design, ensuring its scientific rigor and rationality. Subsequently, a pilot survey was conducted with 50 faculty members and students to test the comprehensibility and measurement stability of the questionnaire. Given that China, as the most populous country in the world, possesses a vast market potential (49), Beijing, as the economic, technological, and healthcare hub of China, is at the forefront of medical technology innovation and application. The city has a high level of education among its residents, strong health management awareness, and a high acceptance of emerging medical technologies, including WMD. Therefore, this study selected Beijing residents who have previously used WMD as research participants to enhance its representativeness and external validity.

This study distributed the questionnaire through a social networking online platform, with respondents filling it out anonymously. The survey instructions explicitly informed participants about the study’s purpose and content, assuring them that no personal privacy information would be collected and that all data would be used exclusively for research analysis. To ensure linguistic accuracy and consistency in the questionnaire, three bilingual linguists, fluent in both Chinese and English, were employed by the research team to carry out a back-translation, ensuring alignment in meaning between the two versions. The survey was conducted from December 13, 2024, to December 25, 2024. Each valid respondent received a thank-you reward of 5 RMB upon completing the questionnaire. Of the 411 questionnaires collected, 362 valid responses were retained after removing incomplete and invalid entries during the data cleaning process.

### 4.2 Demographic characteristics of respondents

The demographic characteristics of the survey respondents are summarized in Table 1. Among the participants, 171 (46.96%) were male and 192 (53.04%) were female. The majority of respondents were in the 18–29 age range (215, 59.39%), followed by individuals aged 30–39 (84, 23.2%), 40–49 (49, 13.54%), and 50–59 (14, 3.87%). Most respondents had attained a university-level education (238, 65.75%).

**Table 1:** Demographic characteristics of the survey respondents.

Participant Demographics	*N* = 362	%
Gender		
Female	170	46.96
Male	192	53.04
Age (in years)		
18 ~ 29	215	59.39
30 ~ 39	84	23.20
40 ~ 49	49	13.54
50 ~ 59	14	3.87
Education		
High school or below	89	24.59
College Degree	238	65.75
Master’s or Doctorate degree	35	9.66

### 4.3 Analytical method

First, this study conducted demographic analysis using SPSS, followed by hypothesis testing with SmartPLS 4.0. There are two main types of Structural Equation Modeling (SEM): Covariance-Based SEM (CB-SEM) and Variance-Based SEM (VB-SEM). VB-SEM is generally more suitable for analyzing small sample sizes, non-normally distributed data, and models involving more than six variables (50). Given that this study involved a relatively small sample and included six variables, multivariate normality was assessed using an online calculator (https://webpower.psychstat.org/, accessed on March 16, 2025). The results showed significant multivariate skewness (Mardia’s *β* = 38.105, *p* < 0.01) and non-significant multivariate kurtosis (Mardia’s *β* = 531.24, *p* > 0.05), indicating a violation of multivariate normality (51). Therefore, VB-SEM was adopted to test the proposed hypotheses in this study.

### 4.4 Assessment of bias

To control for potential non-response bias arising from demographic characteristics, this study employed a paired samples t-test to examine differences in key variables between the first 30 and last 30 respondents during the questionnaire collection process. The statistical findings demonstrated no substantial differences in the key research variables between the two groups, confirming that non-response bias was adequately managed.

Common method bias is one of the most prevalent issues in questionnaire-based research, and scholars typically use the full collinearity variance inflation factor (FLL-VIF) approach to detect it in PLS-SEM studies. The data analysis results indicate that the VIF values for all measurement indicators are below the critical threshold of 5 (as shown in the Appendix Table A1), suggesting that common method bias is not a significant threat to the reliability and validity of this study.

### 4.5 Measurement model test

As shown in Table 2, this study systematically evaluated the measurement model using indicators such as outer loadings, discriminant validity, composite reliability (CR), and average variance extracted (AVE). The results of the measurement model assessment indicate that the CR values for all latent variables exceed the acceptable threshold of 0.7, while the Cronbach’s *α* coefficients also surpass the critical value of 0.7, demonstrating good internal consistency of the research data (52). Additionally, all latent variables report AVE values above 0.5, with measurement item outer loadings surpassing the recommended benchmark of 0.7. These indicators collectively confirm that the measurement model exhibits strong convergent validity (52).

**Table 2:** Reliability and validity coefficients for constructs.

Latent variable	Item	Outer loadings	Cronbach’s α	CR	AVE
PE	PE1	0.851	0.907	0.935	0.782
	PE2	0.902			
	PE3	0.915			
	PE4	0.868			
EE	EE1	0.841	0.899	0.930	0.768
	EE2	0.861			
	EE3	0.890			
	EE4	0.912			
SI	SI1	0.916	0.861	0.915	0.783
	SI2	0.931			
	SI3	0.803			
FC	FC1	0.867	0.894	0.926	0.758
	FC2	0.875			
	FC3	0.854			
	FC4	0.887			
GM	GM1	0.876	0.895	0.927	0.761
	GM2	0.870			
	GM3	0.871			
	GM4	0.872			
CI	CI1	0.882	0.860	0.914	0.781
	CI2	0.877			
	CI3	0.892			

As illustrated in Table 3, discriminant validity in this study was evaluated using both the Fornell–Larcker criterion and the Heterotrait–Monotrait Ratio of Correlations (HTMT). According to the standards proposed by Hair et al. (50), the HTMT values among all latent variables are below the critical threshold of 0.85, and the square root of the average variance extracted (AVE) for each latent variable is greater than its correlation coefficients with other latent variables (52). Based on these findings, the measurement model in this study demonstrates satisfactory reliability, convergent validity, and discriminant validity, meeting the fundamental requirements for structural equation modeling analysis (52).

**Table 3:** Discriminant validity.

Fornell–Larcker Criterion						
	CI	EE	FC	GM	PE	SI
CI	0.884					
EE	0.423	0.876				
FC	0.379	0.364	0.871			
GM	0.377	0.351	0.321	0.872		
PE	0.416	0.448	0.445	0.436	0.884	
SI	0.387	0.357	0.324	0.320	0.312	0.885

Heterotrait–Monotrait Ratio						
	CI	EE	FC	GM	PE	SI
CI						
EE	0.481					
FC	0.431	0.405				
GM	0.432	0.393	0.357			
PE	0.467	0.496	0.495	0.484		
SI	0.445	0.406	0.373	0.366	0.354	

This study assessed model fit using the standardized root mean square residual (SRMR). The obtained SRMR value of 0.045 falls within the acceptable range, as it is below the 0.08 threshold. Therefore, the model fit is considered satisfactory (53).

This study utilizes the SRMR as a metric to evaluate the model’s goodness of fit. The findings indicate that the model’s SRMR value is 0.045, falling below the recommended threshold of 0.08 (53). This result indicates that the theoretical model aligns well with the empirical data and that the model’s specification is appropriate. As a comprehensive measure of model residuals, a smaller SRMR value signifies a stronger fit of the model to the data. Thus, the findings of this study validate the reasonableness of the theoretical model (53).

Additionally, this study conducted a diagnostic test for potential multicollinearity issues. By calculating the VIF, it was found that all variables had VIF values below the critical threshold of 5 (52). This result indicates that there is no severe multicollinearity among the explanatory variables, ensuring that multicollinearity does not significantly affect the parameter estimation results. This diagnostic result further enhances the credibility and robustness of the study’s conclusions.

### 4.6 Structural model test

*R*^2^ refers to the predictive power or explanatory capability of a structural equation model. In this study, the *R*^2^ value for continuance intention was 0.457, indicating that the research model accounts for a portion of the variance in users’ continuance intention. Specifically, to test the theoretical model, this study employed PLS-SEM 4.0, and the results of the path coefficients and hypothesis tests are provided in Table 4. The findings indicate that PE (*β* = 0.148, *p* < 0.005), EE (*β* = 0.161, *p* < 0.005), and SI (*β* = 0.187, *p* < 0.001) all exert a significant positive impact on users’ intention to continue usage, thereby supporting hypotheses H1, H2, and H3. Nevertheless, the impact of FC on continued usage intention was found to be statistically non-significant (*β* = 0.073, n.s.), and thus, hypothesis H4 was not supported. Notably, gamification design demonstrated a significant positive impact on continued usage intention (*β* = 0.144, *p* < 0.005), which confirms hypothesis H5. Regarding control variables, only the education level variable exhibited a significant positive influence on the intention to continue using WMD (*β* = 0.369, *p* < 0.001), while other demographic variables showed no significant effects. Overall, most of the theoretical hypotheses proposed in this study were empirically supported.

**Table 4:** Assessment of the structural model.

Hypothesis	*β*	STDEV	*T* Statistics	*p*-values
H1: PE → CI	0.148	0.046	3.207	0.001
H2: EE → CI	0.161	0.049	3.264	0.001
H3: SI → CI	0.187	0.044	4.282	0.000
H4: FC → CI	0.073	0.044	1.649	0.099
H5: GM → CI	0.144	0.045	3.157	0.002
Gender → CI	−0.136	0.083	1.649	0.099
Age → CI	0.000	0.039	0.011	0.992
Edu → CI	0.369	0.038	9.600	0.000

Finally, this study employed the Stone-Geisser *Q*^2^ test (54, 55) to evaluate the predictive relevance of the model. The results show that the *Q*^2^_predict value for continued usage intention is 0.338, which significantly exceeds the critical threshold of 0, indicating that the theoretical model has strong predictive validity (56). Additionally, the model’s GOF was assessed by calculating the fit index and using the SRMR as a supplementary measure. In this study, the SRMR value is 0.045, which falls below the suggested threshold of 0.08 (57), confirming that the model fits the empirical data effectively. Taken together, the *Q*^2^_predict and SRMR indices confirm that the model specification is reasonable and possesses acceptable explanatory and predictive power.

## 5 Discussion and conclusion

### 5.1 Key findings

With the widespread application of WMD in the healthcare sector, their advantages in providing convenient and efficient services are gradually becoming apparent. However, some users abandon usage due to a lack of interest or feelings of boredom. Given the significant effectiveness of gamification design in enhancing user interest and motivation, this study innovatively integrates gamification variables into the UTAUT model, constructing a comprehensive theoretical analysis framework. This extended model aims to explore, from a multidimensional perspective, the key factors influencing users’ continued use of medical wearable devices. By applying traditional technology acceptance theories to healthcare, this research also presents a new theoretical approach to understanding the role of gamification design in shaping user behavioral intentions.

First, the results reveal that PE significantly and positively influences users’ intention to continue using. This result is consistent with prior research on mobile health applications (40) and health management-oriented WeChat official accounts (41). This indicates that when users view wearable devices as helpful for health management, their intention to continue using them increases significantly. This finding further validates the importance of the practical utility of device functions in users’ decisions to continue usage.

The substantial positive impact of EE on users’ continued usage intention is consistent with prior studies on mobile health services (29, 40). This indicates that, in healthcare contexts, users are more likely to choose technologies or devices that are easy to operate and have user-friendly interfaces, making it easier for them to manage their health.

The SI significantly and positively affects users’ continued intention to use. These findings support prior studies on wearable device adoption among older adults (43). This finding suggests that users’ decision-making processes are significantly influenced by the opinions of significant others or groups, consistent with the social psychological theory that individual behavior aligns with social expectations (42).

Contrary to initial assumptions, the effect of FC on users’ continued usage intention was not statistically significant. This differs from the findings of Lin and Lee (45) on green finance technology and also contrasts with the research of Dwivedi et al. (46) on mobile health services. One possible explanation is that current wearable devices primarily involve the collection of personal data and health monitoring functions (13), which are connected to smartphones for data sharing, location tracking, and communication features. While this technological characteristic enhances the functionality of the devices, it may also raise concerns among users about data privacy and information leakage, thereby reducing the positive impact of FC.

The strong positive impact of gamification on users’ continued usage intention aligns with previous research on fitness applications (48). This suggests that gamification elements like points, badges, and leaderboards can transform monotonous health monitoring and exercise tracking into more engaging and interactive experiences, significantly enhancing users’ interest and willingness to use the app.

Additionally, the empirical results indicate that education level is the only control variable that significantly and positively influences users’ intention to continue using WMD. One possible explanation is that users with higher education levels generally exhibit greater technological acceptance and a higher propensity to explore new technologies (58).

### 5.2 Theoretical contributions

This study systematically examines the factors affecting users’ intention to continuously use WMD from a theoretical perspective, offering two key contributions. First, by adopting the UTAUT model as the theoretical framework, it investigates the influence of PE, EE, SI, and FC on users’ continued usage intention. These results further confirm the relevance of the UTAUT model in explaining WMD adoption. This analysis not only provides theoretical support for understanding user behavior regarding wearable devices but also extends the contextual applicability of the UTAUT model. Second, this study introduces gamification as an additional variable, demonstrating its significant role in the use of WMD. This finding enriches the existing literature on user behavior related to wearable devices and fills a research gap regarding gamification in this field. By revealing the positive impact of gamification elements—such as points, reward mechanisms, and leaderboards—on users’ continued usage intention, this study offers new perspectives and insights for theoretical advancements in related fields.

### 5.3 Practical contributions

This study translates its findings into the following practical recommendations for optimizing the design and marketing of WMD.

First, optimizing gamification design can enhance users’ expected benefits of wearable devices in health management by incorporating clear, quantifiable goal-setting and real-time feedback mechanisms. This approach helps users intuitively perceive their health improvement progress.

Second, improving user experience is essential. Simplifying the device’s user interface and reducing operational complexity ensure that users can seamlessly complete health-related tasks. Additionally, offering personalized health recommendations and dynamically adjusting task difficulty based on user needs allow users to achieve their goals within an acceptable range, thereby increasing their willingness for continued use.

Third, enhancing social interaction can further boost engagement. Introducing team-based task modules, such as collaborative and competitive modes, encourages users to participate in health challenges with family and friends, fostering a sense of involvement and strengthening user retention.

### 5.4 Limitations and future research

Despite its theoretical and practical contributions, this study has certain limitations that should be recognized. The primary limitation lies in the geographic constraint of the sample: data collection for this study was primarily concentrated in the Beijing area, which may cause the research findings to be influenced by regional economic development levels and cultural backgrounds. Specifically, as Beijing is China’s political, economic, and cultural center, its residents’ technology acceptance, purchasing power, and health awareness may differ systematically from those in other regions. Therefore, future research is encouraged to broaden the geographical scope of the sample and implement a comparative design across multiple regions and cultural contexts to assess the generalizability and robustness of the findings. Additionally, cross-cultural comparative research can help deepen the understanding of how regional cultural differences affect users’ technology acceptance behaviors.

Secondly, the cross-sectional nature of this study constrains the ability to capture temporal variations in users’ intention to continue using wearable devices. Future studies may consider utilizing a longitudinal design to better understand long-term behavioral trends.

An additional limitation of this research is its sample coverage, which does not include older adult users aged 60 and older. This exclusion is due to the fact that this age group often faces significant issues related to the “digital divide.” Such sample selection bias may result in a lack of sufficient explanatory power when applying the findings to the older adult population. Therefore, future studies should target the older adult user group and investigate in greater detail their behavioral patterns, psychological challenges, and the factors influencing their adoption of WMD.

Finally, this study primarily relies on the UTAUT model to explain users’ usage intentions. However, the UTAUT model has certain limitations when applied to complex contexts. Future research could enhance the theoretical framework by incorporating new variables or boundary conditions—such as personality traits and technology trust—to improve the explanatory power of user behavior.

## Data Availability

The original contributions presented in the study are included in the article/supplementary material, further inquiries can be directed to the corresponding author.

## Appendix

**Table A1:** Variable items utilized.

Factors	Serial num.	Item	Reference
Performance expectancy (PE)	PE1	I believe wearable devices are very useful for collecting health information.	(20)
	PE2	Using wearable devices allows me to quickly access health information.	
	PE3	Using wearable devices provides me with the health information I need.	
	PE4	If I use wearable devices, I will increase my opportunities to manage my health.	
Effort expectancy (EE)	EE1	I can quickly and easily use wearable devices.	
	EE2	I can proficiently use wearable devices and the health information they provide.	
	EE3	I believe wearable devices easy to use.	
	EE4	I believe learning to operate wearable devices is easy for me.	
Social influence (SI)	SI1	The people around me support my use of wearable devices.	
	SI2	The people who are important to me believe that I should use wearable devices.	
	SI3	The people around me believe that I should use wearable devices.	
Facilitating conditions (FC)	FC1	I have the resources required to use wearable devices.	
	FC2	I possess the necessary knowledge to use wearable devices.	
	FC3	If I encounter difficulties while using wearable devices, someone will be available.	
	FC4	Wearable devices are compatible with other systems, making them convenient for everyday use.	
Gamification (GM)	GM1	When I use wearable medical devices, I feel like I’m participating in a health management game.	(19)
	GM2	The badges or rewards earned from using wearable medical devices make me feel like I’ve completed several health challenge tasks.	
	GM3	I enjoy using wearable medical devices because they allow me to accumulate health points or achievements, much like playing a game.	
	GM4	Each time I complete a health goal or challenge set by the wearable medical device, my experience is similar to completing a task in a game.	
Continued Intention (CI)	CI1	I would strongly recommend others to use wearable devices.	(41)
	CI2	I plan to continue purchasing the next generation of wearable devices in the future.	
	CI3	I plan to continue using wearable devices.	
